# Mast cells contribute to double-stranded RNA-induced augmentation of airway eosinophilia in a murine model of asthma

**DOI:** 10.1186/1465-9921-14-28

**Published:** 2013-03-04

**Authors:** Keiko Kan-o, Yuko Matsunaga, Satoru Fukuyama, Atsushi Moriwaki, Hiroko Hirai-Kitajima, Takehiko Yokomizo, Kosuke Aritake, Yoshihiro Urade, Yoichi Nakanishi, Hiromasa Inoue, Koichiro Matsumoto

**Affiliations:** 1 Research Institute for Diseases of the Chest, Graduate School of Medical Sciences, Kyushu University, Fukuoka 812-8582, Japan; 2 Department of Biochemistry, Juntendo University School of Medicine, Tokyo 113-8431, Japan; 3 Department of Medical Biochemistry, Graduate School of Medical Sciences, Kyushu University, Fukuoka 812-8582, Japan; 4 Department of Molecular Behavioral Biology, Osaka Bioscience Institute, Osaka 565-0874, Japan; 5 Department of Pulmonary Medicine, Graduate School of Medical and Dental Sciences, Kagoshima University, Kagoshima 890-8520, Japan

**Keywords:** Mast cells, Virus, Double-stranded RNA, Allergic asthma, Knockout mice

## Abstract

**Background:**

Clinical studies showed the contribution of viral infection to the development of asthma. Although mast cells have multiple roles in the pathogenesis of allergic asthma, their role of in the virus-associated pathogenesis of asthma remains unknown. Most respiratory viruses generate double-stranded (ds) RNA during their replication. dsRNA provokes innate immune responses. We recently showed that an administration of polyinocinic polycytidilic acid (poly IC), a mimetic of viral dsRNA, during allergen sensitization augments airway eosinophilia and hyperresponsiveness in mice via enhanced production of IL-13.

**Methods:**

The effect of poly IC on allergen-induced airway eosinophilia was investigated for mast cell-conserved Kit^+/+^ mice and -deficient Kit^W^/Kit^W-v^ mice. The outcome of mast cell reconstitution was further investigated.

**Results:**

Airway eosinophilia and IL-13 production were augmented by poly IC in Kit^+/+^ mice but not in Kit^W^/Kit^W-v^ mice. When Kit^W^/Kit^W-v^ mice were reconstituted with bone marrow-derived mast cells (BMMCs), the augmentation was restored. The augmentation was not induced in the mice systemically deficient for TIR domain-containing adaptor-inducing IFN-β (TRIF) or interferon regulatory factor (IRF)-3, both mediate dsRNA-triggered innate immune responses. The augmentation was, however, restored in Kit^W^/Kit^W-v^ mice reconstituted with TRIF-deficient or IRF-3-deficient BMMCs. Although leukotriene B_4_ and prostaglandin D_2_ are major lipid mediators released from activated mast cells, no their contribution was shown to the dsRNA-induced augmentation of airway eosinophilia.

**Conclusions:**

We conclude that mast cells contribute to dsRNA-induced augmentation of allergic airway inflammation without requiring direct activation of mast cells with dsRNA or involvement of leukotriene B_4_ or prostaglandin D_2_.

## Background

The pathogenesis of asthma is frequently associated with airway viral infection. The respiratory sincytial virus, the rhinovirus, and the parainfluenzae virus are reported to contribute to the development of asthma and its acute exacerbation [1,2]. These viruses have single-stranded RNA as their own genome and then generate double-stranded (ds)RNA following infection to their host cells, as an intermediate for replication. Given that dsRNA, longer than 30 base pairs, provokes innate immune responses in mammalian cells, it is a rational approach to target dsRNA for elucidating the common mechanisms of the virus-associated pathogenesis of asthma. We have shown that a low-dose (10 μg/mouse) administration of polyinocinic polycytidilic acid (poly IC), a mimetic of viral dsRNA, during allergen sensitization in mice markedly augments airway eosinophilia and airway hyperresponsiveness (AHR), cardinal phenotypes of allergic asthma [3].

It is well known that mast cells have multiple roles in the pathogenesis of allergic diseases including asthma. Mast cells activated by an allergen/IgE/ FcεR1 cross-linking produce a variety of chemical mediators, chemokines, and cytokines. These compounds mediate an immediate allergic response and the subsequent adaptive immune responses. In addition, mast cells are highly effective sentinels and have been shown to take part in innate immune responses to a variety of pathogens [4,5]. Although accumulating knowledge leads to a hypothesis that mast cells play a pivotal role in the virus-associated pathogenesis of asthma, there has been no experimental evidence to support the above hypothesis. In the present study, we sought to investigate whether mast cells contribute or not to the dsRNA-induced augmentation of asthma phenotype. To this end, we examined the effect of poly IC on an asthma phenotype for mast cell-conserved and -deficient mice and further investigated the outcome of mast cell reconstitution.

## Methods

### Preparation of poly IC

Poly IC (Sigma Aldrich) was dissolved into physiological saline at the final concentration of 100 μg/ml and tested for endotoxin activity by the Limulus HS-T single Test™ (Wako Pure Chemical) with a resolution limit of 0.008 EU (endotoxin units)/ml. The activity of endotoxin was under the detection limit.

### Animals

All experimental procedures were approved by the animal research ethics committee of Kyushu University (reference number: A23-048-1). BALB/c and C57BL/6 mice were purchased from Charles River Japan. Mast cell-deficient (Kit^W^/Kit^W-v^) and mast cell-conserved (Kit^+/+^) mice were purchased from SLC Japan. TRIF^−/−^and IPS-1^−/−^mice on a C57BL/6 background kindly provided by Dr. Shizuo Akira (the Research Institute for Microbial Diseases, Osaka University, Osaka, Japan). IRF-3^−/−^mice on a C57BL/6 background were kindly provided by Tadatsugu Taniguchi (the Department of Immunology, Graduate School of Medicine and Faculty of Medicine, University of Tokyo, Japan). BLT-1^−/−^ mice on a BALB/c background were generated at the Department of Biochemistry, Faculty of Medicine, Kyushu University (Fukuoka, Japan). Hematopoietic PGD_2_ synthase (hPGD_2_S)^−/−^mice on a C57BL/6 background were generated at the Osaka Bioscience Institute (Osaka, Japan). Mice were housed under specific pathogen-free conditions until 6–7 wk of age.

### Sensitization and challenge

Mice were sensitized by an i.p. injection of 10 μg of OVA (Sigma-Aldrich) and 0.3 mg of Al(OH)_3_ (SERVA Electrophoresis) on days 1 and 14 and challenged with 1% OVA in saline mist for 20 min on days 26, 27, and 28, as described previously [3]. Outcome measurements were conducted on day 30 (2 wk interval protocol). Subgroups of Kit^W^/Kit^W-v^ and Kit^+/+^ mice were sensitized on days 1 and 14 and challenged with OVA on days 54, 55, and 56, and measurements were conducted on day 58 (6 wk interval protocol). Mice received an i.p. injection of 10 μg of poly IC 1 h before each OVA sensitization. Mice receiving physiological saline served as controls. For hPGDS inhibition, mice received an administration of 30 mg/kg of HQL-79, an orally selective inhibitor of hPGD_2_S (Cayman-Chemical) or its vehicle solution 1 h before each poly IC treatment. To assess the acute effect of dsRNA, naive mice were sacrificed and their blood was sampled 5 h after a single injection with poly IC.

### Reconstitution of mast cells

Bone marrow-derived mast cells (BMMCs) were generated by cultivation of bone marrow cells from C57BL/6 mice, TRIF^−/−^mice, IRF-3^−/−^mice, or hPGDS^−/−^ mice in the presence of recombinant murine IL-3 at 5 ng/ml. Cells were maintained in an Iscove modified Dulbecco medium (IMDM, PAA) containing 10% FCS (Sigma-Aldrich), 50 mM β-mercaptoethanol, nonessential amino acid, 2 mM L-glutamine, 100 U/ml penicillin, 100 mg/ml streptomycin, and 1 mM sodium pyruvate (all from Gibco BRL). After 4wk of culture, BMMCs represented more than 98% of the total cells, according to a flow cytometric analysis of the expression of CD117 (c-Kit) and FcεR1. Seven week-old Kit^W^/Kit^W-v^ mice were reconstituted with an i.p. injection of 6 × 10^6^ BMMCs and processed for OVA-sensitization/challenge 5 wk after the transfer. The reconstitution of mast cells in the peritoneum was confirmed by flow cytometric detection of GFP-positive cells in the peritoneal lavage fluid of the mice following transfer of BMMCs from systemic GFP-labeled C57BL/6 mice. The reconstitution of mast cells in the lung was confirmed by microscopic detection of toluidine blue-stained mast cells in the airway mucosal tissues of the mice following transfer of BMMCs.

### Measurement of airway hyperresponsiveness (AHR)

Mice were anesthetized with a mixture of ketamine and sodium pentobarbital i.p., and their tracheas were cannulated via tracheostomy. Animals were ventilated to measure airway responsiveness to acetylcholine aerosol, as described previously [6]. The data were expressed as the provocative concentration 200 (PC_200_), i.e., the concentration at which airway pressure was 200% of its baseline value. The values of PC_200_ were expressed as log (PC_200_ × 100).

### Bronchoalveolar lavage

Immediately after blood sampling, mice were exanguinated and their lungs were lavaged with 1 ml of physiological saline via a tracheal cannula. Cell counts were performed as previously described [6]. Samples were centrifuged at 2000 rpm for 10 min, and the supernatants were collected for cytokine ELISA.

### Determination of cytokine concentration

IL-13 in the supernatants of bronchoalveolar lavage fluid (BALF) and IL-6 and IFN-β in the serum were quantified using ELISA kits (BioSource, or R&D Systems).

### Collection of lung cells

Lungs were cut and minced. Complete RPMI 1640 (Gibco BRL) containing 10% FCS (Sigma-Aldrich), 100 U/ml penicillin, and 100 mg/ml streptomycin was added to the minced lungs and incubated for 60 min. The samples were filtered through a cell strainer (BD Falcon). The single-cell suspensions were washed with complete RPMI 1640, and the erythrocytes were lysed with an NH_4_Cl-Tris buffer. Cells were suspended in complete RPMI.

### Intracellular cytokine flow cytometry

Whole lung cells (4 × 10^6^ cells/well) were stimulated with plate-coated anti-mouse CD3 mAb (clone: 145-2C11) at 5 μg/ml plus soluble anti-mouse CD28 mAb (clone: 37.51) at 2 μg/ml for 48 hr. Golgi-Stop (BD Biosciences) was added for the last 6 h of stimulation. The unadherent cells were harvested and preincubated with Fc Block (BD biosciences) and surface staining was performed using peridinin chlorophyll protein (PerCP)-labeled anti-mouse CD4 (clone: RM4-5) or anti-mouse CD8α (clone: 53–6.7) and allophycocyanin-labeled anti-mouse CD3 (clone: 145-2C11) mAbs. Intracellular staining was performed using a commercially available kit (eBioscience). The cells were fixed with a fixation buffer for 20 min. After a wash, cells were suspended in a permeabilization buffer with 5% normal rat serum (COSMO BIO) for 10 min, and biotinylated anti-mouse IL-13 Ab or control IgG (R&D Systems) was added for 30 min. After a wash with a permeabilization buffer, cells were incubated in the presence of phycoerythrin (PE)-labeled streptavidin (BD Biosciences) for 30 min. After washes, samples were assessed using a FACSCalibur (BD Biosciences). All antibodies, except for anti-IL-13, were purchased from BD Bioscineces.

### Data analysis

Values are expressed as the mean ± SEM. Parametric data were analyzed using the unpaired *t* test or an ANOVA with Bonferroni’s correction. Non-parametric data were analyzed using the Mann–Whitney *U* test or the Kruskal-Wallis test. P-values less than 0.05 were accepted as statistically significant.

## Results

### Mast cells contribute to dsRNA-induced augmentation of airway eosinophilia and IL-13 production

**Figure 1:**
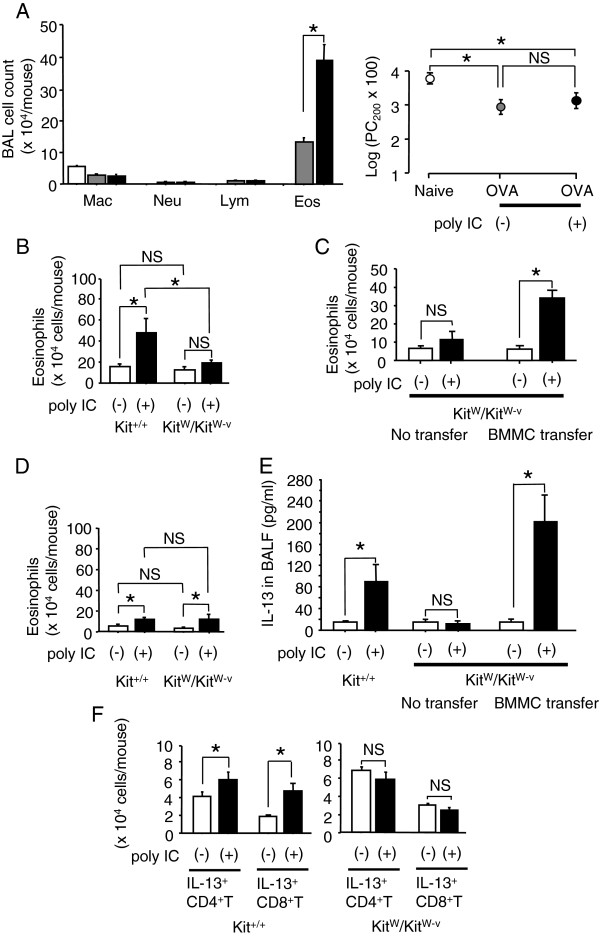
**Effect of poly IC on allergen-induced airway eosinophilia in mast cell-conserved and –deficient mice.** (**A**) C57BL/6 mice were treated with poly IC (black bar and circle) or saline (gray bar and circle) one hour before each sensitization with OVA. Non-sensitized/challenged mice served as naive controls (white bar and circle). Two weeks later, mice were challenged with OVA, and the mice were challenged with OVA, and the eosinophilia in BALF and the airway hyperresponsiveness were measured. The data of airway hyperresponsiveness were expressed as the provocative concentration 200 (PC_200_). The values of PC_200_ were expressed as log (PC_200_ × 100). (**B**) Mast cell-conserved Kit^+/+^ mice or deficient Kit^W^/Kit^W-v^ mice were treated with poly IC or saline before sensitization. Two weeks later, the mice were challenged with OVA, and the eosinophilia in BALF was measured. (**C**) Kit^W^/Kit^W-v^ mice were transferred with or without BMMCs from C57BL/6 mice. Five weeks later, the mice were treated with poly IC or saline before sensitization. Two weeks later, mice were challenged with OVA, and the eosinophilia in BALF was measured. (**D**) Kit^W^/Kit^W-v^ mice were transferred with or without BMMCs from C57BL/6 mice. Five weeks later, mice were treated with poly IC or saline before. Six weeks later, mice were challenged with OVA, and the eosinophilia in BALF was measured. (**E**) Kit^+/+^ mice, Kit^W^/Kit^W-v^ mice, and BMMC-transferred Kit^W^/Kit^W-v^ mice were treated with poly IC or saline before sensitization. Two weeks later, the mice were challenged with OVA, and IL-13 was measured by ELISA. (**F**) Kit^+/+^ mice and Kit^W^/Kit^W-v^ mice were treated with poly IC or saline before OVA sensitization. Two weeks later, mice were challenged with OVA, and IL-13-producing CD4^+^ and CD8^+^T-cell subsets in the lungs were enumerated. Data are expressed as the means ± SEM. Each group consisted of five to nine mice. *P < 0.05 between the indicated two groups. NS means statistically no significant.

In our previous study, an administration of poly IC (10 μg/mouse) during OVA sensitization augmented airway eosinophilia and AHR in OVA-challenged BALB/c mice [3]. These augmented asthma phenotypes were associated with enhanced production of IL-13. Given that Kit^+/+^ mice and Kit^W^/Kit^W-v^ mice are generated on the WB × C57B6 F1 background, the effect of poly IC on the asthma phenotypes was examined for C57BL/6 mice. When the mice were treated with poly IC 1 h before each sensitization with OVA (Figure 1A), the eosinophilia in BALF after OVA inhalation challenge was augmented significantly more than those in saline-treated mice. On the other hand, AHR was not affected by the treatment with poly IC. Hence, the effect of poly IC on asthma phenotype was evaluated by eosinophilia in BALF for the subsequent experiments. The eosinophilia in BALF was significantly augmented by poly IC in mast cell-conserved Kit^+/+^ mice but not in mast cell-deficient Kit^W^/Kit^W-v^ mice (Figure 1B). When Kit^W^/Kit^W-v^ mice were reconstituted with BMMCs from C57BL/6 mice in advance, the augmentation of eosinophilia in BALF was restored (Figure 1C). Of note, eosinophilia in BALF was significantly augmented not only in Kit^+/+^ mice but also in Kit^W^/Kit^W-v^ mice when a longer interval than that described above, 6 wks versus 2 wks after the second sensitization, had been used prior to the OVA challenge (Figure 1D). This finding indicates that mast cells might not be essential for the augmentation of asthma phenotype but strongly promote it. The concentration of IL-13 in BALF in poly IC-treated Kit^+/+^ mice was significantly higher than that in saline-treated Kit^+/+^ mice, while the concentration of IL-13 in poly IC-treated Kit^W^/Kit^W-v^ mice was not different from that in saline-treated Kit^W^/Kit^W-v^ mice (Figure 1E). After the reconstitution with BMMCs, the concentration of IL-13 in poly IC-treated Kit^W^/Kit^W-v^ mice was significantly higher than that in saline-treated Kit^W^/Kit^W-v^ mice. In Kit^+/+^ mice, the numbers of IL-13-producing CD4^+^ and CD8^+^ T cells in the lungs of poly IC-treated mice were significantly larger than those of saline-treated mice (Figure 1F). In Kit^W^/Kit^W-v^ mice, however, the numbers of IL-13-producing CD4^+^ and CD8^+^ T cells were comparable between poly IC-treated mice and saline-treated mice. These results suggest that mast cells contribute to dsRNA-induced augmentation of airway eosinophilia and production of IL-13.

### The TRIF-IRF-3 pathway is essential for dsRNA-induced augmentation of airway eosinophilia

**Figure 2:**
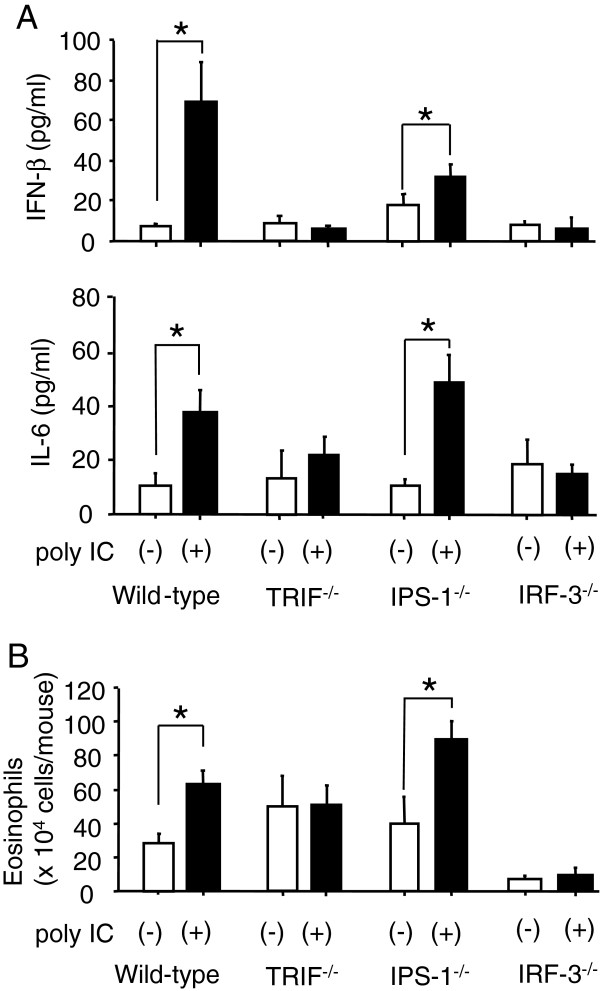
**Role of TRIF, IPS-1, and IRF-3 in poly IC-induced augmentation of airway eosinophilia.** (**A**) Naive C57BL/6 mice, TRIF^−/−^mice, IPS-1^−/−^mice, and IRF-3^−/−^mice were treated with poly IC. The sera were collected five hours after poly IC, and the IFN-β and IL-6 contents were then measured by ELISA. Each group consisted of five to nine mice. (**B**) C57BL/6 mice, TRIF^−/−^mice, IPS-1^−/−^mice, and IRF-3^−/−^mice were treated with poly IC or saline alone before each OVA sensitization. Two weeks later, mice were challenged with OVA, and the eosinophilia in BALF was measured. Each group consisted of five to nine mice. Data are expressed as the means ± SEM. *P < 0.05 between the indicated two groups.

In the innate immune systems, dsRNA is recognized via pattern recognition receptors (PRRs), such as TLR3 and the family of RNA helicase, namely the retinoic acid-inducible gene I (RIG-I) and melanoma differentiation-associated gene 5 (Mda-5) [7-10]. TLR3 recognizes dsRNA in the endsome and initiates signaling through an adaptor, the Toll/IL-1R domain-containing adaptor inducing IFN-β (TRIF) (also known as TICAM-1) [11,12]. RIG-I and Mda5 recognize dsRNA in the cytoplasm and initiates signaling through IFN-β promoter stimulator 1 (IPS-1) (also known as MAVS/Cardif/VISA) [13-16]. The dsRNA/PRRs interaction initiates cascades to produce type-I IFNs and proinflammatory cytokines including IL-6 [17]. Both TRIF and IPS-1 share interferon regulatory factor (IRF)-3 as their downstream transcriptional factor for the production of type-I IFNs [18]. The effects of poly IC on the innate immune responses were compared among TRIF^−/−^mice, IPS-1^−/−^mice, IRF-3^−/−^mice, and wild-type C57BL/6 mice. The concentrations of IFN-β and IL-6 in the sera were assessed 5 h after a single i.p. injection of poly IC to their naive mice (Figure 2A). The injection significantly increased the concentrations of IFN-β and IL-6 in wild-type mice and IPS-1^−/−^mice but not in TRIF^−/−^mice or IRF-3^−/−^mice. Next, the effect of poly IC on airway eosinophilia was evaluated. In wild-type mice and IPS-1^−/−^mice, the poly IC treatment significantly augmented the eosinophilia in BALF, but it did not in TRIF^−/−^mice or IRF-3^−/−^mice (Figure 2B), suggesting that the TRIF-IRF-3 pathway is essential for the poly IC-induced augmentation of airway eosinophilia.

### Direct activation of mast cells with dsRNA is not required for augmentation of airway eosinophilia

**Figure 3:**
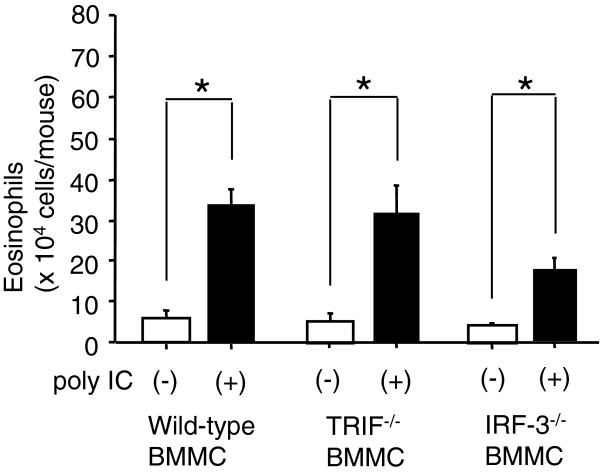
**Effect of TRIF/IRF-3 deficiency in mast cells on poly IC-induced augmentation of airway eosinophilia.** Kit^W^/Kit^W-v^ mice were transferred with BMMCs from C57BL/6 mice, TRIF^−/−^mice, or IRF-3^−/−^mice. Five weeks after the transfer, mice were treated with poly IC or saline alone before each OVA sensitization. Two weeks later, the mice were challenged with OVA, and the eosinophilia in BALF was measured. Each group consisted of five to eight mice. Data are expressed as the means ± SEM. *P < 0.05 between the indicated two groups.

Given the essential role of the TRIF-IRF-3 pathway in the poly IC-induced augmentation, we sought to explore whether this pathway needs to work in mast cells. Kit^W^/Kit^W-v^ mice were reconstituted with BMMCs from TRIF^−/−^mice, IRF-3^−/−^mice or wild-type mice and then treated with poly IC during sensitization (Figure 3). Following the OVA challenge, the augmentation of eosinophilia in BALF was restored not only in the mice reconstituted with BMMCs from wild-type mice but also in those with BMMCs from TRIF^−/−^mice or IRF-3^−/−^mice. Hence, direct activation of mast cells with poly IC via the TRIF-IRF-3 pathway is not required for the augmentation.

### dsRNA-induced augmentation of airway eosinophilia is independent of mast cell-derived prostaglandin D_2_ or leukotriene B_4_

**Figure 4:**
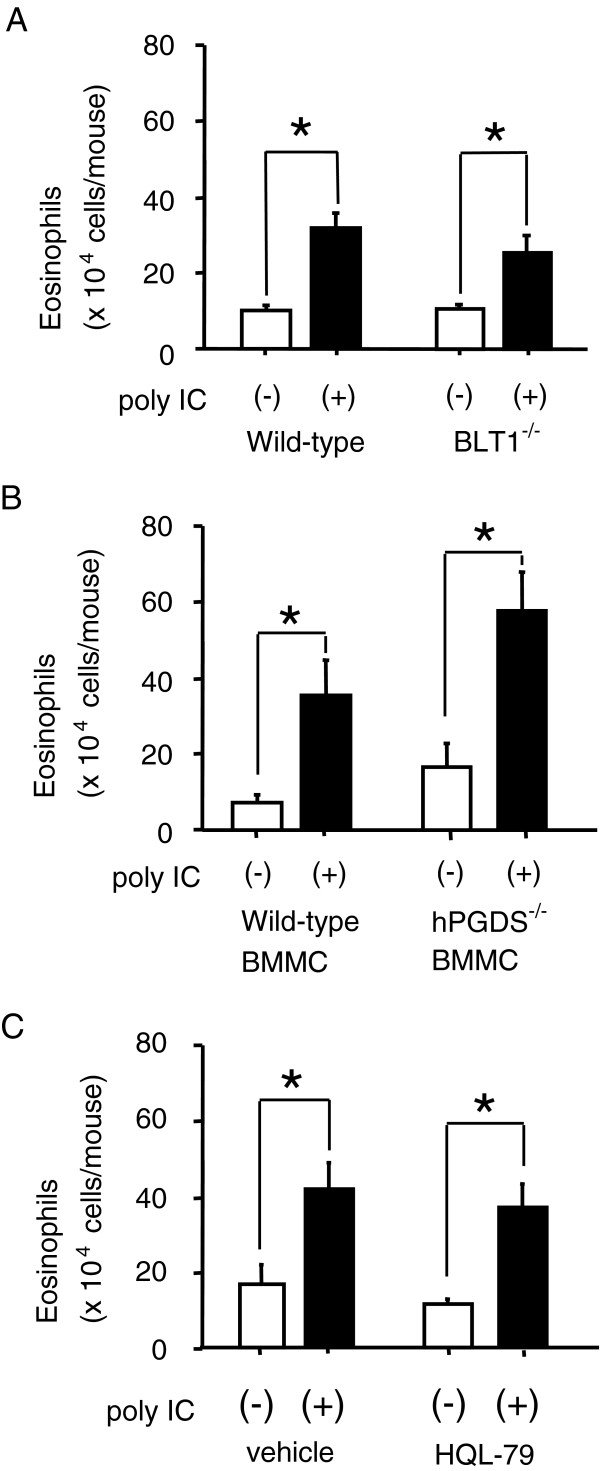
**No involvement of prostaglandin D**_**2**_**- or leukotriene B**_**4 **_**on poly IC-induced augmentation of airway eosinophilia.** The eosinophilia in BALF after OVA challenge was measured for the mice sensitized with OVA in the following conditions (**A** to **C**). OVA challenge was performed two weeks after the last OVA sensitization. (**A**) Wild-type BALB/c mice and BLT-1^−/−^ mice were treated with poly IC or saline alone before each OVA sensitization. (**B**) Kit^W^/Kit^W-v^ mice were transferred with BMMCs from wild-type C57BL/6 mice or hPGD_2_S^−/−^mice. Five weeks after the transfer, mice were treated with poly IC or saline alone before each OVA sensitization. (**C**) BALB/c mice were treated with poly IC or saline alone before each OVA sensitization. HQL-79, a selective inhibitor of hPGD_2_S, was administered just before each poly IC treatment. Data are expressed as the means ± SEM. Each group consisted of six mice. *P < 0.05 between the indicated two groups.

Previous studies showed that mast cells induce the recruitment of IL-13-producing T cells into the airway via releasing leukotriene B_4_ (LTB_4_), a potent chemoattractant for several subsets of T cells by its binding to the high-affinity receptor, BLT_1_[19,20]. In BLT_1_^−/−^ mice, however, the eosinophilia in BALF was significantly augmented by poly IC similarly as it was in wild-type mice (Figure 4A). Several studies have shown that prostaglandin D_2_ (PGD_2_) and its receptors DP and CRTH2 are involved in the pathogenesis of asthma [21-23]. PGD_2_ is a major prostanoid being released from mast cells, which is dependent on the activation of hPGD_2_S [24]. To elucidate whether the poly IC-induced augmentation of airway eosinophilia depends on a mechanism via mast cell-derived PGD_2_, the effect of poly IC was examined for Kit^W^/Kit^W-v^ mice that had been reconstituted with BMMCs from hPGD_2_S^−/−^mice (Figure 4B) [25]. The poly IC treatment significantly augmented the eosinophilia in BALF in the mice reconstituted with hPGD_2_S^−/−^mice-derived mast cells, as it did in those with wild-type mice-derived mast cells. Next, the effect of the poly IC was tested in wild-type mice that received HQL-79, an orally selective inhibitor of hPGD_2_S [26], before each OVA sensitization (Figure 4C). The administration of HQL-79 failed to prevent the augmentation of the eosinophilia in BALF. Taken together, poly IC-induced augmentation of asthma eosinophilia may be independent of mast cell-derived PGD_2_ or LTB_4_.

## Discussion

In the present study, the mice were sensitized with OVA emulsified with alum. A previous study showed that mast cells contributed to the OVA-induced asthma phenotype that developed in mice sensitized with OVA without alum, but not that induced in mice sensitized with OVA with alum [27]. Indeed, the magnitude of airway eosinophilia in Kit^W^/Kit^W-v^ mice was comparable with that in Kit^+/+^ mice in the absence of poly IC treatment. In the presence of poly IC, eosinophilia, IL-13 in BALF, and the numbers of IL-13-producing CD4^+^ and CD8^+^ T cells were augmented in Kit^+/+^ mice but not in Kit^W^/Kit^W-v^ mice. After mast cell reconstitution, eosinophilia and IL-13 in BALF were augmented by poly IC treatment even in Kit^W^/Kit^W-v^ mice. These findings indicate that mast cells may specifically contribute to dsRNA-induced augmentation of airway eosinophilia via amplifying the production of IL-13. The limitation of this study is that we could not clarify when mast cells played their promoting role, whether in sensitization phase or challenge phase. No difference in the effect of poly IC on airway eosinophilia between Kit^+/+^ and Kit^W^/Kit^W-v^ mice in 6 wk interval protocol made it difficult to investigate the effect of BMMC transfer after the sensitization phase on asthma phenotype. According to this limitation, the subsequent examinations were designed in consideration with both cases.

A mast cell is a highly effective sentinel and has been shown to take part in innate immune responses to a variety of pathogens [4,5]. Human and murine mast cells express TLR3, which are capable of producing a broad spectrum of mediators in response to dsRNA [28,29]. A previous study reported that CD8^+^ T-cell recruitment induced by an i.p. injection of poly IC is impaired in Kit^W^/Kit^W-v^ mice with compared to Kit^+/+^ mice and that poly IC-stimulated BMMCs have a chemoattractant activity for CD8^+ ^T cells *in vitro*[30]. We have confirmed that stimulation of BMMCs with poly IC at 3 μg/ml for 5 h significantly enhanced the production of MIP-1α, MIP-1β, and eotaxin (unpublished observation). MIP-1α and MIP-1β preferentially attract CD8^+^ and CD4^+^T cells, respectively [31,32]. However, the augmentation of airway eosinophilia was restored in the mice reconstituted with BMMCs from TRIF^−/−^mice or IRF-3^−/−^mice. These results suggest that direct activation of mast cells with dsRNA is not required for the augmentation if they play a role in sensitization phase. Allergen uptake by local dendritic cells triggers their migration to draining lymph nodes for antigen presentation. Several investigators showed that the migration of dendritic cell subsets into draining lymph nodes are partially dependent on mast cells and associated compounds, including histamine, IL-6, and TNF-α [33,34]. These indirect processes might be involved in the dsRNA-induced augmentation of airway eosinophilia. The determination of underlying mechanisms awaits further investigation.

The role of mast cells in challenge phase is tightly associated with the adaptive immune responses. A subpopulation of Th2 cells preferentially expresses CRTH2, a PGD_2_ receptor mediating chemotaxis [35]. PGD_2_ induces the production of macrophage-derived chemokine (MDC) from airway epithelium, which can recruit CCR4-expressing Th2 cells in mice [36]. In addition, effector CD8^+^ T cells preferentially express a chemotactic receptor, BLT_1_[37,38]. Given that mast cells activated by an IgE-dependent mechanism produce ample of PGD_2_ and LTB_4_, it is plausible that mast cells in the airways may be a major recruiter for Th2 and effector CD8^+^ T cells [39]. Several investigators have shown that BLT_1_ is required for effector CD8^+^ T cell-mediated, mast cell-dependent AHR and inflammation [19,20,40]. However, poly IC treatment augmented airway eosinophilia under a mast cell-derived PGD_2_-deficient or a BLT_1_-deficient condition, suggesting that neither mast cell-derived PGD_2_ nor BLT_1_ is responsible for the dsRNA-induced augmentation of airway eosinophilia. There is another receptor for LTB_4_, namely BLT_2_. We have recently shown that BLT_2_ negatively regulates airway eosinophilia via suppressing the activation of IL-13-producing CD4^+^ T cell in OVA-sensitized/challenged mice [41]. This opposing effect makes it unlikely that BLT_2_ is associated with dsRNA-induced augmentation of airway eosinophilia.

Contrasting to our previous study using BALB/c mice [3], AHR was not augmented by the treatment with poly IC. It is well known that C57BL/6 mice show blunt AHR compared to BALB/c or A/J mice. We confirmed that poly IC treatment significantly augmented IL-13 in BALF even in OVA-sensitized/challenged C57BL/6 mice (unpublished observation). We previously demonstrated that intratracheal administration of IL-13 failed to induce AHR in naïve C57BL/6 mice, whereas the same treatment induced AHR in naïve A/J mice [42]. The difference in the contribution of IL-13 to AHR might account for the lack of augmentation in C57BL/6 mice.

The methodology using intraperitoneal injection of poly IC might limit the interpretation of the results from this study since it induced mild but substantial immune responses. Indeed, the concentrations of IFN-β and IL-6 in the serum were increased 5 h after the injection of poly IC in naïve mice. With this regard, alternative interpretation would be the reflection of physiological aspects of viral infection in the hosts. To gain convincing evidence, application of intranasal or intratracheal administration of poly IC for airway sensitization models is a future challenge.

## Conclusions

We conclude that mast cells contribute to dsRNA-induced augmentation of allergic airway inflammation without requiring direct activation of mast cells with dsRNA or involvement of leukotriene B_4_ or prostaglandin D_2_. Further elucidation of their molecular process will help understand the mechanism underlying the interaction between a viral infection and the pathogenesis of asthma.

## Abbreviations

dsRNA: Double-stranded RNA; poly IC: Polyinocinic polycytidilic acid; BMMCs: Bone marrow-derived mast cells; TRIF: Toll/IL-1R domain-containing adaptor inducing IFN-β; IRF-3: Interferon regulatory factor 3; AHR: Airway hyper-responsiveness; PC200: Provocative concentration 200; hPGD2S: Hematopoietic prostaglandin D_2_ synthase; BALF: Bronchoalveolar lavage fluid; RIG-I: Retinoic acid-inducible gene I; Mda-5: Melanoma differentiation-associated gene 5; IPS-1: IFN-β promoter stimulator 1

## Competing interests

All authors have no conflict of interest.

## Authors’ contributions

KK prepared BMMCs, performed the *in vivo* experiments, did cell differentials and ELISA, and wrote the manuscript. YM prepared BMMCs and did histological staining. SF prepared the protocol of BMMC transfer. AM and HH did cell differentials and ELISA. TY prepared BLT^−/−^mice. KA and YU prepared hPGD_2_S^−/−^mice. YN and HI participated in interpretation of data and manuscript writing. KM designed the study, performed the *in vivo* experiments, did cell differentials, and wrote the manuscript. All authors have read and approved the final manuscript.
